# Acute Pancreatitis: A Narrative Review

**DOI:** 10.7759/cureus.99233

**Published:** 2025-12-14

**Authors:** Emmanuel M Sindato

**Affiliations:** 1 Internal Medicine, Gastroenterology and Hepatology, School of Medicine and Dentistry, The University of Dodoma, Dodoma, TZA

**Keywords:** acute pancreatitis, aetiology, management, prevalence, review

## Abstract

Acute pancreatitis is an increasingly prevalent gastrointestinal condition worldwide with substantial morbidity and mortality. This narrative review summarises the epidemiology and contemporary management of acute pancreatitis. A literature search was conducted in May 2025 to identify studies published from inception to May 2025 in MEDLINE/PubMed, Web of Science, and Scopus. Electronic searches were supplemented by manual screening of reference lists from key articles identified via Google Scholar using the following key terms: “acute pancreatitis, prevalence, epidemiology, aetiology and management” For PubMed, the Medical Subject Headings (MeSH) terms were used based on the research question. Boolean operators were employed to fine-tune the search terms. There were no language restrictions. The diagnostic criteria for acute pancreatitis were aligned with the American College of Gastroenterology guidelines for the management of acute pancreatitis. After screening abstracts and full texts, 31 articles were included in this review. Acute pancreatitis is still a major health concern, with the burden increasing globally, mainly attributed to the increased burden of obesity and gallstones. The most common etiologies of acute pancreatitis are ascribed to biliary disease, alcohol consumption and hypertriglyceridemia. Acute pancreatitis is associated with substantial morbidity and mortality in its severe form, underscoring the need for timely diagnosis and appropriate management to improve outcomes.

## Introduction and background

Acute pancreatitis is an inflammatory condition of the pancreas characterised by severe acute upper abdominal pain and elevated serum levels of pancreatic enzymes. The underlying pathophysiologic process of acute pancreatitis is the autodigestion of the pancreatic parenchyma by pancreatic enzymes; the evolving inflammatory process can be either localised or systemic with organ dysfunction [1-3]. Acute pancreatitis is one of the most common conditions of the gastrointestinal tract leading to hospital admission [1-4]. Globally, the burden of acute pancreatitis is on the rise [5-7]. Severe acute pancreatitis carries a substantial morbidity and mortality, underscoring the need for clinicians to recognise that acute pancreatitis is heterogeneous, progressing differently among patients and is often unpredictable [2]. This narrative review summarises the epidemiology and contemporary management of acute pancreatitis.

## Review

Methods

A literature search was conducted on May 2025 to identify studies published from inception to May 2025 in MEDLINE/PubMed, Web of Science, Scopus and supplemented electronic searches with manual searches of the reference lists of key articles from Google Scholar using the following key terms: “acute pancreatitis, prevalence, epidemiology, aetiology and management” For PubMed, the Medical Subject Headings (MeSH) terms were used based on the research question. Boolean operators were employed to fine-tune the search terms. There were no language restrictions. The diagnostic criteria for acute pancreatitis were aligned with the American College of Gastroenterology (ACG) Guidelines of Management of Acute Pancreatitis [2]. After screening abstracts and full texts, 31 articles were included in this review.

Epidemiology

Globally, the incidence of acute pancreatitis varies between five and 80 per 100,000 population [5,7,8]. The wide variation is attributed to differences in risk factors, capacity of healthcare systems, and population characteristics across regions. The highest burden is documented in the US [5]. The incidence in Europe is about 17.5 cases per 100,000 people [5,7,8]. Leading etiological factors vary with geographical location; for instance, in the US, the leading cause of acute pancreatitis is alcohol, while in Europe, gallstones are the most common documented cause of acute pancreatitis [5,8]. There is data paucity in Asia and sub-Saharan Africa, and the real burden and pattern in these regions is poorly characterised.

The median age for acute pancreatitis depends on the underlying aetiology. Epidemiological studies show that the peak age for alcohol-induced pancreatitis is between 30 to 40 years, whereas gallstone-related cases are more frequent in the elderly [8].

Sex related demographics depict that alcohol-pancreatitis is more common in males than females, and females tend to have gallstones as the most common aetiology [9].

Epidemiological projection models show that the incidence of acute pancreatitis is on the rise globally due to increased rates of obesity and gallstones [8]. Acute pancreatitis mortality is as high as 17% in necrotising acute pancreatitis and about 5% in oedematous interstitial acute pancreatitis [3,4,10].

Aetiology

Globally, gallstones represent the most common cause of acute pancreatitis. Literature shows that the burden of gallstones in causing acute pancreatitis ranges from 35% to 80% [3,4,8], with geographical variation, with the highest burden being in developed countries. Occult microlithiasis presumably is the main cause of idiopathic acute pancreatitis [11]. The mechanism involved is likely due to stones obstructing the ampulla, leading to an increase in pancreatic ductal pressure and potentially triggering acinar cell injury [11]. The lifetime risk of developing gallstones acute pancreatitis among patients with gallstones is about 6% [5,6,12].

Although the global alcohol consumption has slightly decreased over the past decade, from 5.7 litres per capita in 2010 to 5.5 litres in 2019 [13], alcohol is the second most common cause of acute pancreatitis, particularly chronic alcohol consumption, and it is responsible for about 35% of cases [3,8]. The risk of developing alcohol induced acute pancreatitis is estimated to be about 10% among individuals with chronic alcohol use disorder, although habitual drinking and binge episodes have also been implicated in alcohol related acute pancreatitis [14-16]. The implicated mechanisms include alcohol inducing premature activation of pancreatic enzymes; furthermore, alcohol promotes the formation of protein plugs, which obstruct the pancreatic flow [14,15].

Among medical procedures associated with acute pancreatitis, retrograde cholangiopancreatography (ERCP) is the leading cause, averaging up to 10% of cases among patients undergoing ERCP [17]. Post-ERCP risk is reported to be up to 5% and as high as 40% among individuals with low risk and high risk, respectively [17]. Risk factors associated with post-ERCP acute pancreatitis include: ERCP intended to treat sphincter of Oddi dysfunction, young age, female gender, number of attempts to cannulate the papilla, inexperienced operator and poor emptying of the pancreatic duct after opacification [17].

Another important metabolic condition causing acute pancreatitis is hypertriglyceridemia. Serum triglyceride levels above 11 mmol/L can cause attacks of acute pancreatitis; it is responsible for up to 14% cases of acute pancreatitis [18]. Hypertriglyceridemia-induced pancreatitis should be sought among patients with high risks, particularly individuals with primary lipoprotein disorders and those with cardio-metabolic conditions such as obesity and diabetes.

Viral infections have also been associated with acute pancreatitis. Common viruses involved include mumps, coxsackievirus, cytomegalovirus, hepatitis viruses, and Epstein-Barr virus [19]. These pathogens may directly infect pancreatic tissue or provoke immune responses that may lead to acute pancreatitis [19]. Although viral-acute pancreatitis is rare, and the exact pathophysiological process is not well documented, viral aetiology should be considered in patients with abdominal pain and elevated pancreatic enzymes, especially if a recent viral illness is reported.

Hereditary factors also contribute to acute pancreatitis, with genetically predisposed individuals leading to premature trypsin activation. Genetic mutations associated with acute pancreatitis include: gain-of-function mutations in the PRSS1 gene, mutations in the CFTR gene have been associated with an autosomal recessively inherited pancreatitis, mutations in the SPINK1 and mutations in the CTRC gene [20].

Drug-induced acute pancreatitis is a rare phenomenon, but an important cause to consider, particularly when other causes have been excluded, and the patient is under medication. In most cases, the prognosis of drug-induced pancreatitis is excellent and has a low mortality rate. The implicated mechanisms involve immunologic reactions, toxic metabolites, and increased viscosity of pancreatic juice [21,22]. Table 1 summarises the causes of acute pancreatitis.

**Table 1:** Aetiologies of acute pancreatitis ERCP; Endoscopic Retrograde Cholangiopancreatography, SOD; sphincter of Oddi

Aetiology	Occurrence	Risk factors
Gallstones	40 to 70% [3,11,23]	About 3 to 7% of patients with gallstones may develop gallstone pancreatitis
Alcohol	25 to 35 % [3,14–16]	History of alcohol abuse
Triglycerides	1 to 14% [18]	Serum hypertriglyceridemia above 11.1mmol/L
ERCP	2 to 10% [17]	Patients undergoing ERCP, risk increases with the SOD treatment, young age, female gender and number of attempts to cannulate inexperienced endoscopist, prolonged sphincter of Oddi manipulation
Genetic predisposition	<1% [20]	Mutations in PRSS1, CFTR, SPINK1 and CTRC genes
Drugs: Didanosine Azathioprine 6-mercaptopurine Tetracyclines	<5% [21,22]	History of exposure to offending drug
Viral		Preceding febrile illness

Clinical features/presentation

Physical evaluation is commonly evident with signs of hypovolaemia, tachycardia and tachypnoea. Fever is not uncommon, attributed by either the intense pro-inflammatory cytokines release, in particular interleukin-6, TNF-α and interleukin-1 beta, or may be due to a complicated pancreatitis, such as pancreatic necrosis with or without infection [23].

Abdominal pain is the most common presentation of acute pancreatitis. Typically, the abdominal pain is of severe intensity, sudden onset, located in the epigastric region and left upper quadrant abdominal pain, which is described as dull and steady in nature. Most commonly, the pain radiates to the back, and its intensity tends to increase over time until reaching a constant, severe pain [1-3]. The pain is often accompanied by nausea, vomiting, and anorexia. Diarrhoea can occur in some cases, and pain may improve temporarily when the patient leans forward. These symptoms can be easily misdiagnosed as acute gastritis or peptic ulcer disease, especially among inexperienced clinicians. It is paramount, therefore, to have a higher suspicion index for acute pancreatitis amid this presentation [24]. Among patients with gallstone pancreatitis, the pain is localized and the onset of pain is more rapid, reaching peak intensity in a short time, and history may be noted for recurrent biliary pain. In severe acute pancreatitis, patients can present with pulmonary symptoms, mainly dyspnoea due to diaphragmatic inflammation secondary to pancreatitis, pleural effusions, or acute respiratory distress syndrome [24].

Diagnosis and differential diagnosis

Timely diagnosis of acute pancreatitis is paramount in reducing the morbidity and mortality associated with acute pancreatitis. Diagnosis principally depends on the symptomatology, biochemical profiling and imaging. Most guidelines recommend diagnosing acute pancreatitis depending on abdominal pain with characteristics aligning with acute pancreatitis, serum lipase or amylase levels at least three times the upper limit of the normal and findings of acute pancreatitis on abdominal sonography and/or computed tomography (CT) or magnetic resonance imaging (MRI). Generally, abdominal CT is not indicated for patients with mild pancreatitis and is commonly performed after 72 hours. Contrast-enhanced CT should be performed in patients complicating into systemic illness, septic or those without improvement to exclude acute pancreatitis complications such as peripancreatic collections, necrosis, abscesses, tumour and vascular complications [1]. Magnetic resonance cholangiopancreatography (MRCP) is preferred to diagnose suspected biliary and pancreatic duct causes of acute pancreatitis. Additionally, MRCP will provide radiographic imaging that will inform clinical care and decision to undertake ERCP [1]. ACG guidelines for the management of acute pancreatitis 2024 recommend transabdominal ultrasonography in patients with acute pancreatitis to evaluate for biliary pancreatitis and a repeat ultrasonography if the initial examination is inconclusive. Further recommendations for patients with cryptogenic acute pancreatitis are to repeat abdominal sonography, MRI or endoscopic ultrasound [2]. Table 2 summarises the differentials of acute pancreatitis.

**Table 2:** Common differential diagnosis of acute pancreatitis ALT; alanine aminotransferase, AST; aspartate aminotransferase, CBD; common bile duct, CRP; C-Reactive Protein, CT; computed tomography, PUD; peptic ulcer disease, RUQ; right upper quadrant, ULN; upper limit of normal.

Condition	Clinical presentation	Laboratory tests	Imaging findings
Acute Pancreatitis	Sudden severe epigastric pain radiating to the back, nausea, vomiting, fever	3x ULN elevation of Serum amylase & lipase [1,2], leucocytosis, raised CRP	Presence of focal or diffuse pancreas enlargement, peripancreatic fat stranding, fluid collections on CT
PUD	Epigastric pain related to meals, may improve or worsen with food	Normal amylase/lipase, possible anaemia if bleeding	Normal appearance of pancreas on abdominal imaging. Mucosal defect on endoscopy, free air if perforated
Acute Cholecystitis	RUQ/epigastric pain, fever, Murphy’s sign positive	mild elevations in serum ALT/AST [1] and amylase, hyperbilirubinemia R-factor ≤ 2.0: indicating cholestatic injury	Normal appearance of pancreas on abdominal imaging. Gallbladder wall thickening, pericholecystic stranding, gallstones may be evident
Choledocholithiasis	may have a history of gallstones, RUQ pain	Normal Serum amylase/lipase R-factor ≤ 2.0: indicating cholestatic injury	Normal appearance of pancreas on abdominal imaging. CBD stone on abdominal imaging
Intestinal Obstruction	Crampy abdominal pain, vomiting, distension, obstipation, or constipation	↑ Lactate, leucocytosis, < 3x serum lipase/amylase elevation [1,2]	Normal appearance of pancreas on abdominal imaging. Bowel wall thickening, dilated loops of bowels with air fluid levels
Gastroenteritis	RLQ pain, fever, anorexia	Mild leucocytosis, normal amylase/lipase	
Renal Colic	Severe, colicky flank		Normal appearance of pancreas on abdominal imaging. Identifies renal stones, hydronephrosis
Acute Mesenteric Ischemia	Severe periumbilical abdominal pain, cardio-metabolic risks	Mild serum lipase/amylase elevation	Normal appearance of pancreas on abdominal imaging. focal or segmental bowel wall thickening or intestinal pneumatosis with portal vein gas

Classification of severity

Categorising the acute pancreatitis severity scale is of paramount importance in order to individualise the approach towards management so as to improve morbidity and mortality. Severity classification can either be based on clinical severity assessment or based on morphology following imaging [2,25,26]. Evaluation of acute pancreatitis severity classification aims to identify patients at risk for organ failure or complications who may require intensive monitoring and support, especially within the first 72 hours, and plan a tailored resuscitation plan. Important clinical signs to examine for severity index include pulse less than 40 or more than 150 beats/minute, systolic blood pressure less than 80 mmHg, diastolic blood pressure more than 120 mmHg and respiratory rate more than 35 breaths/minute. Laboratory investigations include CRP, FBP, comprehensive biochemical profile and lactate [2,25,26]. Daily severity evaluation is crucial so as to identify severity grade worsening and to promptly manage as needed. CT imaging is recommended after 48-72 hours if the patient does not improve, as necrosis may not be evident early on until up to 72-96 hours [2]. Severity stratification can be challenging in the first 24 hours and often relies on scoring systems like Systemic Inflammatory Response Syndrome (SIRS) and Acute Physiology and Chronic Health Evaluation II (APACHE II) after 24-48 hours. ICU admission should be considered for patients with severe disease or critical abnormalities in vital signs and laboratory values [2,25,26].

Management and outcomes

The management approach of acute pancreatitis is tailored according to the severity of acute pancreatitis, complications management and the management of underlying predisposing conditions. Timely correct diagnosis, severity grading and identifying underlying aetiologies are paramount in reducing morbidity and mortality. The management plan discussed here is in line with the ACG Guidelines and the World Society of Emergency Surgery (WSES) for the management of acute pancreatitis. Medical treatment focuses on fluid resuscitation, pain management and early enteral feeding. Surgery is indicated in biliary pancreatitis or infected necrotising pancreatitis. Early ERCP should be considered in patients with acute pancreatitis associated with cholangitis or persistent biliary obstruction [1,2,26,27].

Fluids resuscitation

Current acute pancreatitis management guidelines recommend fluid resuscitation with lactated Ringer’s solution over normal saline for initial fluid resuscitation in patients without hypercalcemia, in whom normal saline is the preferred fluid for resuscitation. Current evidence is against the use of hydroxyethyl starch-containing fluids [27]. Guidelines recommend the moderate intravenous hydration approach with an initial 10 mL/kg bolus in patients with hypovolemia, followed by 1.5 mL/kg/hour with goal-directed therapy for fluid management in the first 24 to 48 hours after onset of symptoms [1,27-29]. Close monitoring is crucial, and fluid titration should be tailored based on the volume status, particularly in the first 12, 24, 48, and 72 hours. Appropriate fluid resuscitation is paramount as it is associated with a reduction in morbidity and mortality [1,27-29]. 

Pain management

The approach towards pain management depends on the pain severity scale, categorised as mild pain and moderate to severe pain. For mild pain management, options include weak opioids and acetaminophen. In patients with moderate to severe pain due to acute pancreatitis, the approach is to use strong opioids such as hydromorphone or fentanyl. Adequate pain control is crucial, as uncontrolled pain may compound into haemodynamic instability [1,27].

Feeding

For mild acute pancreatitis, the current evidence shows that early oral feeding (within 24-48 hours) is suggested as tolerated by the patient, moving away from the traditional "nothing-by-mouth" approach [1]. According to the ACG acute pancreatitis management guideline (2024), it is recommended that initial oral feeding begin with a low-fat solid diet rather than a stepwise liquid to solid approach [2]. Early oral feeding in mild and moderately severe acute pancreatitis is safe, without increasing the risk of complications like necrosis or organ failure. Current literature indicates that early oral feeds as tolerated reduce the time to initiate solid feedings, accompanied by fewer sepsis incidences and shorter hospital stays and lower costs. In patients with moderately severe and severe pain due to acute pancreatitis, enteral nutrition (tube feeding via nasogastric or nasojejunal route) is preferred over parenteral nutrition, with the current evidence showing that it is associated with fewer infectious complications. Parenteral nutrition should be avoided unless the enteral route is not possible, not tolerated, or not meeting caloric needs. The tube choice between the nasogastric and nasojejunal route for enteral feeding, the nasogastric tube is preferred due to comparable safety and efficacy, and easier placement. The advantages of enteral feeding include maintaining gut mucosal integrity, preventing bacterial translocation that can lead to infected pancreatic necrosis [28].

Prophylactic antibiotics

While the old approach was to prescribe prophylactic antibiotics, current guidelines are against this practice. Antibiotics are not recommended in patients with acute pancreatitis without infection, regardless of the type (interstitial or necrotising) or disease severity (mild, moderately severe, or severe, and should be prescribed in the setting of confirmed infection. It is also important to note that about 20% of patients with acute pancreatitis will develop extra-pancreatic infection. Thus, empiric antibiotics should be initiated when the clinical judgement warrants, especially in the setting where culture and sensitivity are not readily practical [1,2].

Management of complications

For patients presenting with cholangitis, early ERCP is preferred; ideally should be performed within 24 to 48 hours of admission, among patients with gallstone pancreatitis and cholangitis, in addition to antibiotics and intravenous fluids. Urgent ERCP has been shown to reduce the severity of acute pancreatitis, local and systemic complications and mortality [1,2]. Among patients presenting with gallstone pancreatitis but without cholangitis, ERCP can be deferred, as the literature has shown that urgent ERCP in patients without cholangitis does not significantly reduce pancreatic complications, single organ failure, or mortality, although one trial showed a shorter hospital stay. Cholecystectomy in this situation should be performed after recovery [1,2,26,27]. In the setting of biliary sludge as a cause of acute pancreatitis, laparoscopic cholecystectomy significantly reduces recurrent idiopathic acute pancreatitis, even when endoscopic ultrasonography did not detect small gallstones or sludge [1,2].

Follow-up

Following an episode of acute pancreatitis, patients might experience recurrent spells of acute pancreatitis, particularly if the underlying aetiology is not adequately addressed, such as continued alcohol abuse and hypertriglyceridemia. Pancreatic exocrine and endocrine dysfunction are not uncommon, and may develop in up to 30% of patients; furthermore, another 30% might develop into chronic pancreatitis [27]. A systematic review and meta-analysis study by Huang W et al., 2025, has shown that the risk of developing exocrine pancreatic insufficiency after acute pancreatitis increases by almost two-fold [30]. Screening for exocrine pancreatic insufficiency during follow-up visits should be considered. Population-based matched-cohort studies have shown that, after one episode of acute pancreatitis, the risk of developing pancreatic cancer increases ninefold as compared to individuals without an episode of acute pancreatitis [31]. Therefore, pancreatic cancer surveillance should be considered among patients diagnosed with acute pancreatitis.

## Conclusions

Acute pancreatitis is still a major health concern, with the burden increasing globally, mainly attributed to the increased burden of obesity and gallstones. The most common causes of acute pancreatitis include gallstones, alcohol consumption and hypertriglyceridemia. Severe acute pancreatitis underscores the timely diagnosis and initiation of appropriate management in order to reduce morbidity and mortality. Patients presenting with biliary pancreatitis are subject to surgery to prevent the incidence of recurrent pancreatitis. Early oral feeding is paramount in preventing complications. Prophylactic antibiotics should be avoided; they should preferably be initiated when infection is confirmed. Following an episode of acute pancreatitis, patients are at risk of recurrence, exocrine and endocrine dysfunction, progression to chronic pancreatitis, and increased likelihood of pancreatic cancer, underscoring the need for follow-up screening and surveillance.
